# A data-driven approach for predicting the impact of drugs on the human microbiome

**DOI:** 10.1038/s41467-023-39264-0

**Published:** 2023-06-17

**Authors:** Yadid M. Algavi, Elhanan Borenstein

**Affiliations:** 1 Faculty of Medicine, Tel Aviv University, Tel Aviv, Israel; 2 Blavatnik School of Computer Science, Tel Aviv University, Tel Aviv, Israel; 3 Santa Fe Institute, Santa Fe, NM USA

**Keywords:** Microbiome, Machine learning

## Abstract

Many medications can negatively impact the bacteria residing in our gut, depleting beneficial species, and causing adverse effects. To guide personalized pharmaceutical treatment, a comprehensive understanding of the impact of various drugs on the gut microbiome is needed, yet, to date, experimentally challenging to obtain. Towards this end, we develop a data-driven approach, integrating information about the chemical properties of each drug and the genomic content of each microbe, to systematically predict drug-microbiome interactions. We show that this framework successfully predicts outcomes of in-vitro pairwise drug-microbe experiments, as well as drug-induced microbiome dysbiosis in both animal models and clinical trials. Applying this methodology, we systematically map a large array of interactions between pharmaceuticals and human gut bacteria and demonstrate that medications’ anti-microbial properties are tightly linked to their adverse effects. This computational framework has the potential to unlock the development of personalized medicine and microbiome-based therapeutic approaches, improving outcomes and minimizing side effects.

## Abstract (web summary)

Drugs can impact the gut microbiome. Here, Algavi and Borenstein developed a machine-learning framework that successfully predicts the impact of thousands of drugs on hundreds of gut microbes, explaining drug-induced dysbiosis and side effects.

## Introduction

Our gastrointestinal tract harbors a flourishing and diverse community of microorganisms, collectively known as the human gut microbiome. Over the past decade, we came to appreciate how this microbiome governs individualized responses to diet and susceptibility to a wide array of diseases such as diabetes and cancer^1^. Importantly, however, microbiome research has also revealed complex and bidirectional interactions between the microbiome and numerous pharmaceuticals. On the one hand, many gut-dwelling microbes metabolize drugs, potentially affecting their toxicological, pharmacokinetic, and pharmacodynamic properties^2–6^. On the other hand, many small-molecule drugs alter the taxonomic composition of the microbiome and potentially give rise to various gastrointestinal side effects. Indeed, population-wide case-control studies in the UK and Netherlands have identified many commonly used drugs, including atypical antipsychotics, NSAIDs, and statins, as influential modulators of the intestinal microbiota^7–9^. Additionally, for specific medications, longitudinal clinical studies uncovered temporal variation following drug administration. For example, metformin—an oral glucose-lowering drug used to treat type 2 diabetes—was demonstrated to shift the microbial population in the gut, increasing the prevalence of beneficial short-chain fatty acid-producing species^10^. At the same time, metformin was also shown to increase the abundance of virulent *E. coli* strains that can cause diarrhea, bloating, and nausea— frequent adverse effects in metformin-treated patients. While such clinical studies provide some perspective on drug effects, they cannot be performed on a large number of drugs.

As an alternative approach, a complementary in vitro methodology can be used to explore the potential impact of non-antibiotic drugs on gut microbes. Maier et al.^11^, for example, conducted a high-throughput screen of more than 1000 common drugs against 40 representative gut bacteria under anaerobic conditions. They measured the growth of each species optically over time and showed that 24% of the drugs with human targets inhibit the growth of at least one species. Similarly, others have tested 43 compounds against five different microbial communities and quantified, using mass spectrometry, the absolute bacterial abundance and proteome alterations following drug exposure^12^. This new understanding of how drugs impact the microbiome offers a novel way to improve pharmaceutical treatment as well as minimize side effects^13^. Although such recent studies have cast light on many interactions of interest, a comprehensive and complete understanding of microbiome-drug interactions is still lacking, and the incorporation of pharmacomicrobiomics into clinical practice is accordingly yet out of reach^14^.

To address this challenge, we integrate chemical and microbiological knowledge with a computational, data-driven, systems approach, aiming to predict the impact of a large set of drugs on the growth of microbiome members. Such computational approaches have been successfully applied to predict other types of microbiome-drug relationships (e.g., drug biotransformation^15^), and could thus be similarly effective in facilitating large-scale characterization of drug–microbiome interactions. To demonstrate its conceptually broad applicability, we show that this approach successfully identifies the effect of pharmaceuticals on microbial strains in vitro and can be further applied to predict drug-induced community changes in longitudinal animal models and clinical studies. Moreover, this methodology allows us to systematically map the influence of thousands of drugs on numerous microbial strains, uncover drug and microbial properties that underlie anti-commensal activity, and explore the connection between drug-induced microbiome compositional changes and side effects.

## Results

### Development of a machine learning model for predicting the impact of drugs on the microbiome in vitro

**Fig. 1:**
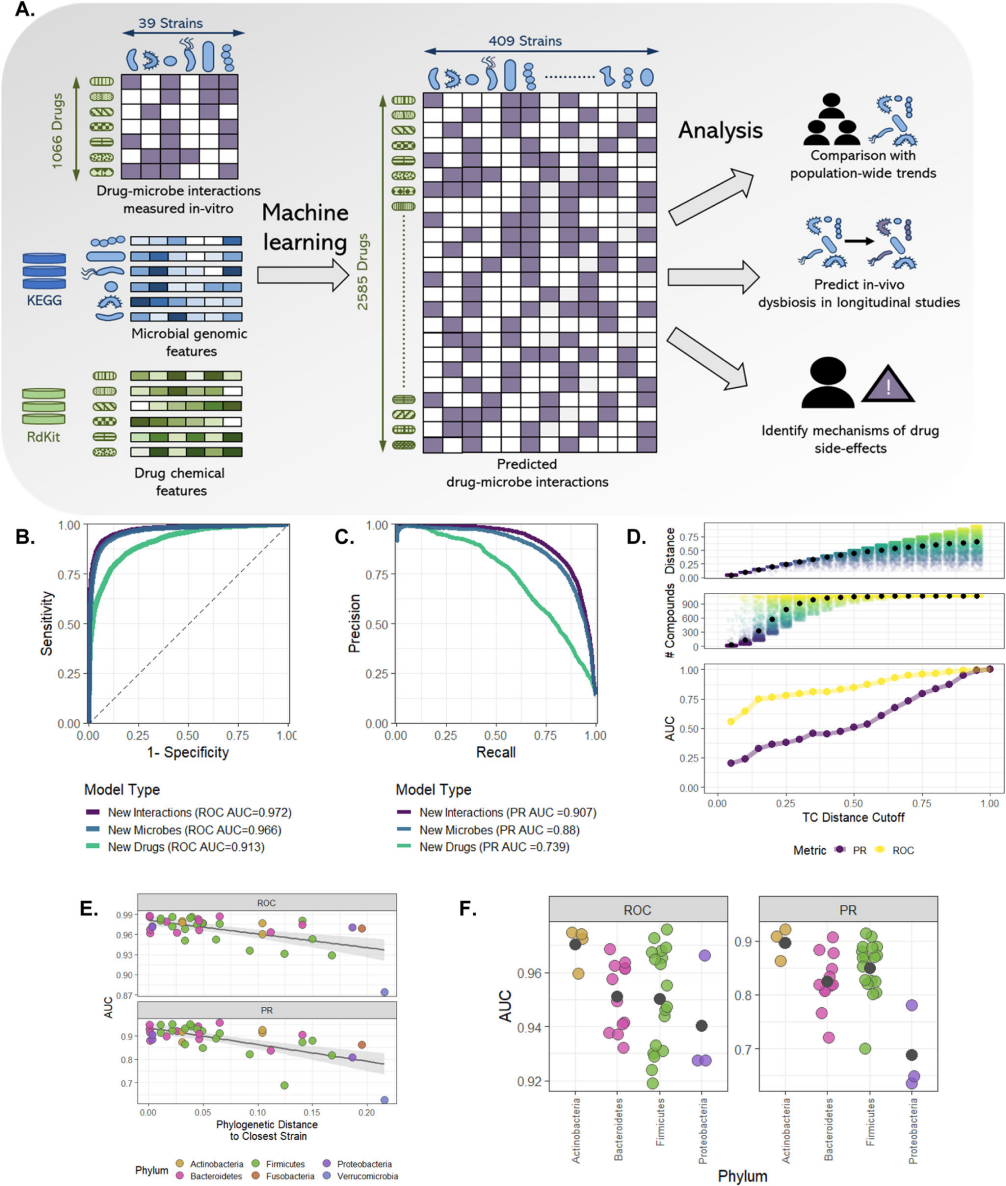
Machine-learning model prediction of in vitro drug–microbe interactions. **A** A scheme of our computational framework. **B** Receiver operating characteristic (ROC) curve for three learning settings: new drug–microbe interactions, new microbes, and new drugs. **C** Precision recall (PR) curve for the three learning settings as in panel **B**. **D** The upper panel displays the distribution of distances from the test compound to the nearest molecule in each cross-validation training set as a function of the TC distance cutoffs. The middle plot shows the distribution of the number of compounds in the training set. The lower panel illustrates the ROC AUC (yellow) and PR AUC (purple) for new drug prediction as a function of the TC distance cutoffs. **E** ROC AUC and PR AUC scores for the leave-one-microbe-out model as a function of phylogenetic distance (with Pearson correlation of −0.574, *p* = 5 × 10^−4^ for ROC AUC and −0.648, *p* = 8.23 × 10^−6^ for PR AUC, error bands show standard error). The color indicates the strain’s phyla. **F** Decrease in ROC AUC and PR AUC score when all strains for the same phylum are removed from the training set. The phyla Verrucomicrobia and Fusobacteria were discarded from this analysis as each contains only a single strain.

We first set out to develop a random forest model that can predict the influence of any drug on any microbial species. Our model takes as input two vectors of features: one representing a specific microbial taxon and the other representing a selected drug—and aims to predict the impact of that drug on that microbe. Specifically, we characterize each microbe by the set of biochemical pathways its genome encodes and each drug by its physical-chemical properties. Overall, our model uses 148 microbial features (describing the number of genes in its genome from each KEGG pathway; Methods), and 92 drug features (properties obtained from the drug’s SMILES representation; Methods). The complete set of features used can be found in Supplementary Data 1. The model then aims to predict a continuous numerical value between 0 and 1 (that we will term throughout the paper as “impact score”), which describes the likelihood that the drug causes growth inhibition (Fig. 1A; see Methods for complete details).

Given this representation, we then trained our model using a large-scale dataset describing a set of in vitro experiments where 40 cultured microbial strains were each exposed to an array of 1197 drugs, determining whether each drug inhibits the growth of each strain (“0”—no effect; “1”—growth inhibition; throughout this work, strains are defined as the specific representatives used in the above in vitro experiment)^11^. We limited the analysis to strains with available genomes (39 out of 40 strains) and compounds included in the DrugBank database (1066 out of 1197, see Methods—Machine-learning model, data, and evaluation). While most drugs were assayed against the complete panel of strains, some of the possible 41,574 combinations (39 strains × 1066 drugs) were missing from the published dataset, resulting in a total of 41,519 drug–microbe interactions. We tested our approach using tenfold cross-validation across this set of interactions. The model demonstrated excellent predictive performance in predicting new drug–microbe interactions in vitro with an area-under-the-receiver-operator curve (ROC AUC) of 0.972 (Fig. 1B). While ROC AUC is clearly an informative metric for assessing the overall predictive power of the model, we further report here (and throughout the paper) the area-under-the-precision-recall curve (PR AUC), which provides valuable information about predictive ability in cases of extensive class imbalance. Indeed, here, even though such class imbalance exists in the dataset (ratio of 1:6.1, in favor of “0” interactions), the model PR AUC performance is 0.907, indicating that the model correctly captures both types of interactions (Fig. 1C). To confirm the robustness of our approach, we conducted 100 iterations of the tenfold cross-validation procedure described above, finding only minimal variance in precision, sensitivity, and specificity across iterations (Supplementary Fig. S1). Similarly, we extensively examined other machine learning models, validated that the results cannot be attributed to statistical noise or artifacts in the data, and systematically benchmarked the model against a naive null model (see Supplementary Text 1).

Since often the impact of a given drug is relatively consistent (i.e., it either impacts most microbes or does not impact most microbes), and hence predicting a specific drug–microbe interaction when the impact of that same drug on other microbes has been used for training may not be challenging, next, we set out to examine the model’s predictive power on new drugs using a leave-one-drug-out approach. We found that while the model performance was slightly lower in these settings, it was still able to successfully predict the impact of new drugs on various microbial strains (ROC AUC of 0.913 and PR AUC of 0.739; Fig. 1B, C). Notably, even after excluding antibiotics and other non-human-targeted compounds, the model can still distinguish between human-targeted drugs with antimicrobial activity and those without (ROC AUC 0.86), suggesting that it does not merely distinguish antibiotics vs. non-antibiotic compounds. Furthermore, to confirm that our predictions are not solely based on identifying chemically similar drugs (which accordingly have similar bioactivity), we evaluate the degree of molecular similarity between each pair of drugs using Tanimoto coefficients (TC)^16^. TC, also known as the Jaccard index, quantifies the similarity between pairs of compounds in the range of 0 (low similarity) to 1 (high similarity) based on their molecular structure. In chemical literature, a TC of 0.85 is commonly considered a good predictor for similar bioactivity. In our dataset of 1066 tested drugs, the mean pairwise TC was 0.20, and the mean TC similarity to the most similar drug was 0.671, suggesting that most drugs have only relatively distantly related drugs in the dataset and hence cannot easily and naively rely on structural similarity for learning. To better estimate the influence of molecular similarity on the model performance, we repeated the leave-one-drug-out approach above while excluding all compounds that are similar to the predicted drug using several similarity thresholds spanning the complete range (1 > TC > 0, Fig. 1D). Indeed, removing all drugs with high structural similarity (according to the above-mentioned cutoff of TC = 0.85), there was only a mild decrease in performance, with a 2.6% decrease in ROC AUC and a 13% decrease in PR AUC. This indicates that the model retains excellent predictive power even when the most relevant examples are removed from the training set. In fact, the performances remained reasonable even at much lower cutoffs, where prediction is performed based on highly different compounds. For example, even when using TC similarity as low as 0.3, which corresponds to having a training set that includes only drugs with very different bioactivity, the model retained a reasonable level of predictive power with a ROC AUC of 0.73 and PR AUC of 0.3.

Further, using an analogous leave-one-microbe-out approach, we inspected the model’s capabilities in predicting the response of new microbial strains. The model demonstrates significant predictive power with a ROC AUC of 0.966 and PR AUC of 0.88 (Fig. 1B, C). Examining the prediction accuracy of our model for strains of various phyla, we further confirm accurate predictions for the two main phyla of the human gut microbiome, Bacteroidetes, and Firmicutes (ROC AUC 0.974, 0.966, PR AUC 0.91, 0.899, respectively). As expected, the model performance improves when phylogenetically similar strains are included in the training set, indicating that phylogenetic similarity translates to related drug response (Fig. 1E). To further determine the robustness of the model, drug response was predicted separately for each microbe when excluding *all* other microbes from the same phylum from the training set. Even under these conditions, performance remains robust with all strains achieving a ROC AUC above 0.92 (Fig. 1F).

Lastly, to further validate our model’s predictions on independent datasets, we evaluated its performances on additional in vitro screening results. First, we utilized data describing 25% inhibitory concentration values (IC25) for 25 human-targeted medications against selected microbial strains^11^. For each drug, we compared our model prediction for that drug’s impact on each strain against the measured IC25. Of the 25 drugs tested, we found a negative correlation between the strain’s impact score and the measured IC25 values in 21 compounds, with FDR-corrected *p* < 0.2 for ten drugs (Spearman correlation test; Supplementary Fig. S2A and Supplementary Data 2). Second, we used an independent dataset describing the impact of 43 drugs on ex-vivo human fecal samples^12^. Evaluating the prediction accuracy of our trained model on this new validation dataset, we found that it exhibits good transferability in predicting specific drug–microbe interactions, with a ROC AUC of 0.70 (Supplementary Fig. S2B). While the prediction accuracy in the second dataset was somewhat lower, it should be noted that both the screening methodology and the drug concentration range used in this second dataset were fundamentally different from those used in the main dataset. Considering these differences, the obtained performances suggest that our model’s predictive power is not database- or technology-specific but potentially universal. We also estimated the predictions on new drugs and new microbes using a leave-one-out approach as before, finding only a minor loss in performance (2–3% decrease in ROC AUC; Supplementary Fig. S2B). These findings confirm that the model successfully predicts drug–microbe interactions on various microbial strains, even when applied to datasets obtained from different experimental setups.

### Structural and microbial features that influence drug impact on the microbiome

**Fig. 2:**
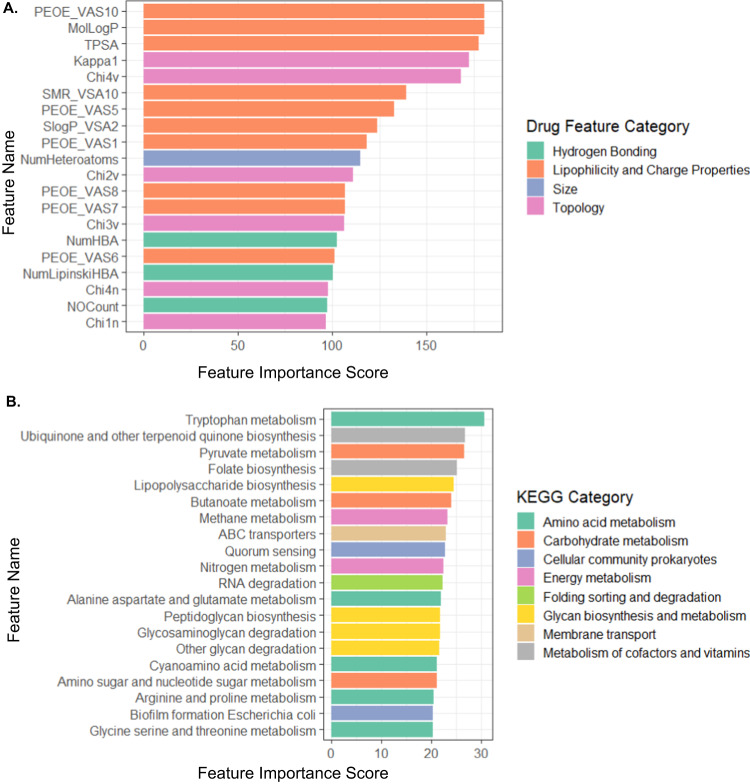
The model’s drug and microbial feature importance scores. **A** The top 20 drug features with the highest feature importance score are colored according to the drug feature category. **B** The top 20 microbial features with the highest feature importance score are colored according to KEGG categories. All features included in these plots are statistically significant (*p* values calculated by permutation importance method, all values with FDR-corrected *p* < 0.05, exact values are provided in Supplementary Data 3).

We next examined the features that contributed most to our model’s predictions to further reveal intriguing and valuable insights into the factors that impact drug–microbe interactions. To this end, we used a permutation importance method to calculate the statistical significance and contribution of each feature^17^. Briefly, this widely used approach estimates the importance of a given feature vector and how different its contribution is from its contribution when the data is shuffled (see Methods for details). We first examined which drug features are most informative for predicting antimicrobial activity. We found that measures of compound lipophilicity (MolLogP) and charge distribution (PEOE) are the most significant contributors to predicting antimicrobial activity (all features with FDR-corrected *p* < 0.05, Fig. 2A and Supplementary Data 3). These are known factors that influence the permeability of bacterial membranes^18,19^. Similarly, surface charge properties (such as total polar surface area—TPSA), hydrogen bonding, and topological features (such as Kappa and Chi connectivity indices) were also found to be contributing to prediction, and are indeed important for binding and passing through porins and other membranous tunnels, with high importance, especially in gram-negative bacteria^18,20^. Likewise, topological drug descriptors of compound shape and geometry (such as Kappa1) were also found to be informative, and indeed such features are known to be relevant for antibiotic accumulation inside bacteria^21^. Additionally, we examined whether the relative importance of drug features varies between different microbes, training the model on one strain at a time, calculating feature significance for each such model, and using a principal component analysis (PCA) to explore variation in these feature importance sets. We found that strain-specific drug feature importance scores cluster according to both phylum and gram stain (Supplementary Fig. S3A, B; PERMANOVA *p* = 1 × 10^−3^ and *p* = 0.017 for phylum and gram stain, respectively). Interestingly though, the main difference in feature importance scores between microbial strains is observed in the topological, charge, and lipophilicity descriptors (Supplementary Fig. S3C), while kappa1, molecular weight, and the number of hydrogen bonding acceptors further differentiate between gram-positive and gram-negative strains (all with FDR *p* < 0.05; *t*-test). Indeed, hydrogen bonding and size have been reported to differ between gram-positive and negative antibacterial compounds^18^. We also examined the feature importance obtained for a model trained only on human-targeted drugs as these drugs, unlike antibiotics, were not optimized for cellular penetrance. Comparison between the two lists of important drug features revealed increased importance of topological features in human-targeted drugs (p = 0.031), with a general positive correlation between the two lists (Pearson correlation 0.523, *p* = 1.7 × 10^−6^).

We further investigated in a similar manner which microbial features contributed most to the prediction of drug–microbe interactions. Interestingly, we found that 54 biochemical pathways from 16 KEGG categories significantly contributed to the model (with FDR *p* < 0.05, Fig. 2B). Significant microbial features include known cellular processes that are essential for antibiotic resistance. For example, indole—one of the main byproducts of tryptophan metabolism, and the top-ranking feature in this list, is known to cause antibiotic tolerance in bacteria^22^. Similarly, another product of tryptophan metabolism, indole-3-acetic acid, is recognized as a defense mechanism against various forms of stress^23,24^. Furthermore, co-factor biosynthesis pathways such as ubiquinone production and folate biosynthesis are known to regulate oxidative stress^25,26^. Not surprisingly, features that encode for membranal structure and transport, lipopolysaccharide biosynthesis, and ABC transporters were also found to have high importance^19^. We also examined potential differences in the abundance of these features across the various phyla (Supplementary Fig. S3D), and indeed, found that many of these features are enriched in members of the more drug-resistance phyla. For example, ABC transporters are more abundant among proteobacteria, whereas tryptophan metabolic capacities are abundant among gram-negative strains. We further found that the microbial features that contribute to the original model and those that contribute to a model trained on only human-targeted drugs are highly correlated (Pearson correlation 0.904, *p* = 2.2 × 10^−16^), suggesting that similar genomic components might be utilized for resistance against antibiotics and non-antibiotic drugs, reinforcing previous findings^11^.

### Predicting drug–microbe interactions on a large scale and identifying determinants of drug impact and microbial sensitivity

Having established and benchmarked our model using available data on a drug–microbe interactions in vitro, we set out to explore the complete landscape of interactions between drugs and members of the human gut microbiota. Toward this aim, we collected all clinically approved small-molecule drugs from the DrugBank database (*n* = 2585 compounds)^27^ and calculated their physio-chemical properties. Similarly, to represent the diversity of the intestinal bacterial community, we used metagenomic data from a representative, healthy western population^28^ (see Methods for details). Notably, although these samples were obtained from a relatively limited population, they are drawn from an extremely well-characterized healthy population of fecal microbiota transplant donors that were not exposed to antibiotics or other drug treatment. We then trained our model on the complete in vitro data described previously and systematically predicted the impact of each of the 2585 drugs on 409 human microbiota members, resulting in a rich catalog of 1,057,265 drug–microbe interactions (Supplementary Data 4). Notably, more than 62% of the drugs and 90% of the microbial taxa have not been tested in vitro before.

**Fig. 3:**
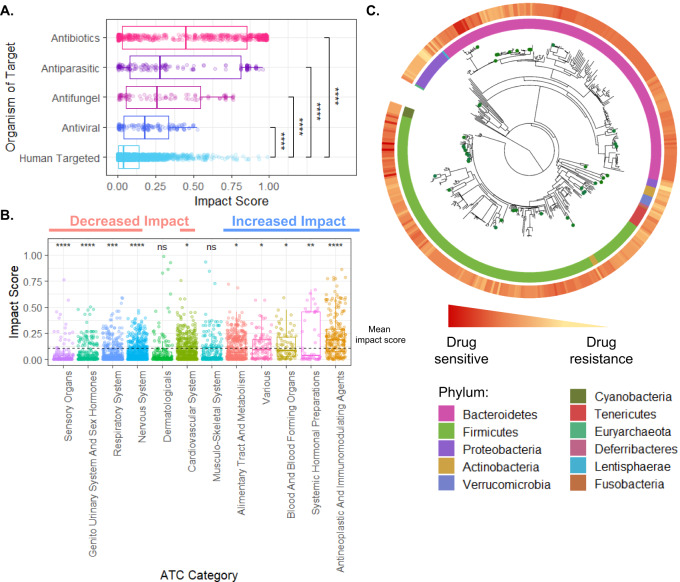
The landscape of drug impact on microbiome members. **A** Box plots describing the difference in impact scores between human target drugs and drugs targeting various microorganisms (pairwise comparison by Wilcoxon rank-sum test). A Kruskal–Wallis test further shows that the differences between ATC categories are statistically significant, *p* = 8.22 × 10^−127^, E^2^ = 0.138. A line across the box indicates the median. The whiskers are lines extending from Q1 and Q3 to endpoints that are defined as the most extreme data points within Q1 − 1.5 × IQR and Q3 + 1.5 × IQR, respectively (Exact values are provided in Supplementary Data 5a). **B** The differences in impact scores between human-targeted drugs according to anatomical therapeutic chemical (ATC) classifications. The symbols above each label indicate the statistical significance in comparison with the mean impact score and the black points indicate the category means (pairwise comparison by Wilcoxon rank-sum test, exact values are provided in Supplementary Data 5b). A Kruskal–Wallis test further shows that the differences between ATC categories are statistically significant, *p* = 2.17 × 10^−64^, E^2^ = 0.102. **C** Phylogenetic tree for 409 microbiome members constructed based on their 16s rRNA gene. The outer circle denotes the drug sensitivity index, and the inner circle denotes the phylum. Taxa marked with green dots are those included in the in vitro screen (*n* = 39). ns not significant; *p < 0.05; **p < 0.01; ***p < 0.001; ****p < 0.0001.

Using this catalog, we first sought to identify patterns in the drug–microbe interaction landscape, and highlight drug- and microbe-specific determinants of such interactions. Examining the predicted impact of each drug on the various taxa revealed that, perhaps not surprisingly, antibiotics cause the most impact on the microbiome. Likewise, pharmaceuticals whose targets are non-human, such as antivirals, antifungals, and antiparasitic drugs, also impact many taxa (Fig. 3A, Kruskal–Wallis test *p* = 8.2 × 10^−127^, E^2^ = 0.138). These compounds were designed to penetrate through the cellular membrane and disrupt vital biochemical pathways that could be shared among organisms. As drugs with overlapping pharmacological and therapeutic properties frequently share bioactivity, it is interesting to compare the antimicrobial properties of human-targeted drugs with different pharmacological classifications. To this end, we obtained the Anatomical Therapeutic Chemical (ATC) classification of each drug in our analysis and focused specifically on the information concerning the physiological system that the drug targets. Indeed, this analysis revealed that drugs with antineoplastic activity show the highest lethality, followed by drugs that act on blood-forming organs, and the alimentary tract. In contrast, medications affecting the sensory, genitourinary, and respiratory systems show the lowest average activity (Fig. 3B, Kruskal–Wallis test, *p* = 2.17 × 10^−64^, E^2^ = 0.102). Considering specifically the top 20 drugs (in terms of their mean impact on microbial taxa) that have not been screened in the original database, we found several compounds with reported in vivo anti-commensal activity such as the immunosuppressants Sirolimus^29^ and Tacrolimus^30^ and the antineoplastic agents Somatostatin^31^ and Eribulin^32^.

Next, we used the above catalog to examine differences in drug sensitivity (which we define as mean impact score across all compounds) across the phylogeny of human gut microbes included in our analysis (Fig. 3C). We found that microbiome members from the Verrucomicrobia and Proteobacteria phyla showed generally higher resistance to drugs. This resistance could be attributed to their rich capacity for drug metabolism and the low permeability of double-layer membranes^33^. Comparing the sensitivity of the two main phyla of the human microbiome, Firmicutes and Bacteroidetes, whose ratio is known to be associated with multiple lifestyles and clinical factors^34^, has also exhibited intriguing patterns. Specifically, while both phyla are predicted to be drug-sensitive, over 80% of the drugs have some specificity for one or the other (Wilcoxon rank-sum test, FDR *p* < 0.05). While antiparasitic, respiratory, and nervous system drugs have a more specific impact on Bacteroidetes, hormonal preparations are more specific toward Firmicutes (Wilcoxon rank-sum test, FDR *p* < 0.05). Moreover, inspecting these dominant phyla, we identified genus-level differences in drug resistance. Specifically, *Firmicutes*, *Roseburia*, and *Blautia* show relatively high sensitivity, whereas *Clostridium* and *Oscillospira* exhibit higher resistance (Supplementary Fig. S4A and Supplementary Data 5). Similarly, in Bacteroidetes, *Alistipes* has the highest resistance, followed by *Bacteroides*, *Parabacteroides*, and *Prevotella* (Supplementary Fig. S4B).

Lastly, it is worth noting, that our computational framework could, in principle, pinpoint potential drugs’ modes of action. As a proof-of-concept, we compared the impact scores of microbes with and without various homologs to recognized drug targets retrieved from the DrugBank database (see Methods for details), identifying 201 putative drug-target interactions (Supplementary Text 2 and Supplementary Data 6). These included both previously known targets, as well as candidates for novel targets, highlighting the potential of our computational model for future studies of drug–microbiome interactions.

### Prediction of in vivo drug-induced dysbiosis

Next, we set out to evaluate the ability of our computational framework to predict the impact of various drugs on the microbiome community in vivo. Extending previous validation^11^, here we aimed to test the impact of specific drugs, both on the much larger set of microbes included in our model’s predictions and in terms of the observed variation in the complete microbiome composition profile following drug administration. Although the intestinal ecological dynamics are far more complex than those of single-strain in vitro experiments, we hypothesized that taxa predicted to be strongly impacted by a given drug would exhibit reduced abundances after pharmacological intervention with that drug. Toward this aim, we collected metagenomic sequences from longitudinal studies in which subjects were sampled before and after pharmaceutical treatment (Supplementary Table 1). This study design allows direct evaluation of the impact of the drug on intestinal bacteria while minimizing other confounding factors that can arise in case-control studies such as inter-individual variability in the microbiome baseline composition. Although these datasets have been produced using 16s rRNA sequencing and hence are limited to analysis at an ASV level, they may still capture broader patterns of modulation of the microbiome induced by drugs. To examine our hypothesis, we compared model predictions of each taxon with the change in their relative abundance after treatment (see Methods).

**Fig. 4:**
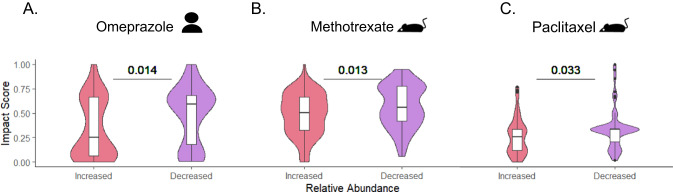
Prediction of in vivo drug-induced dysbiosis. Violin plots illustrating the difference in predicted impact scores between taxa with significantly increased vs. decreased abundance following drug administration of **A** Omeprazole (human clinical trial, *n* = 153 taxa), **B** Paclitaxel (mouse model, *n* = 120 taxa), and **C** Methotrexate (rat model, *n* = 149 taxa). Statistics were calculated by a two-sided Wilcoxon rank-sum test. A line across the box indicates the median. The whiskers are lines extending from Q1 and Q3 to endpoints that are defined as the most extreme data points within Q1 − 1.5 × IQR and Q3 + 1.5 × IQR, respectively.

First, we examined the model’s ability to predict microbiome alteration in human clinical trials. Omeprazole is a common proton-pump inhibitor used in the treatment of gastroesophageal reflux disease and is associated with decreased diversity of intestinal species and increased risk for *Clostridium difficile* infections^35^. Comparing microbiome composition before and after Omeprazole treatment with our model’s predicted impact has demonstrated that our model correctly captures the effect of this drug on the microbiome, assigning a higher impact score to taxa whose relative abundances were reduced following omeprazole administration (Fig. 4A). Importantly, although we trained the model on merely 39 strains, it was able to predict the impact on a microbiome community of 153 different taxa, of which only 15% were tested in vitro against omeprazole while the rest represent novel predictions of the model. Moreover, repeating this analysis while separating taxa tested in vitro from those that were not, we found that the statistical significance reported above is primarily driven by those members of the microbiota not yet tested (Supplementary Fig. S5A). We further applied our model to data available from two pre-clinical animal models treated with Paclitaxel (also known as Taxol), a chemotherapy used to treat various solid cancers^36^, and Methotrexate, the first-line therapy for rheumatoid arthritis^37^. In both cases, our model successfully predicted the drug-induced changes in the gut microbiota (Fig. 4B, C). As for Omeprazole, most of the taxa were previously unscreened in vitro and are driving for statistical significance of these analyses (Supplementary Fig. S5B, C). The additional analysis further confirmed that in all cases above, the results cannot be attributed merely to statistical noise since the randomization of features resulted in total statistical significance loss (Methods; Supplementary Fig. S5D, E).

### Comparing predicted drug–microbe interactions to observed population-wide trends

Following our analysis of longitudinal data from animal models and clinical trials reported above, we sought to explore the agreement between our model’s predictions and patterns observed in broad human populations. Put differently, whereas our analysis of data from longitudinal studies above examined our model using directly measured drug impact (controlling for other confounding factors such as disease, age, etc.), here, we aim to test our predictions in a diverse, large-scale, real-world cohort.

**Fig. 5:**
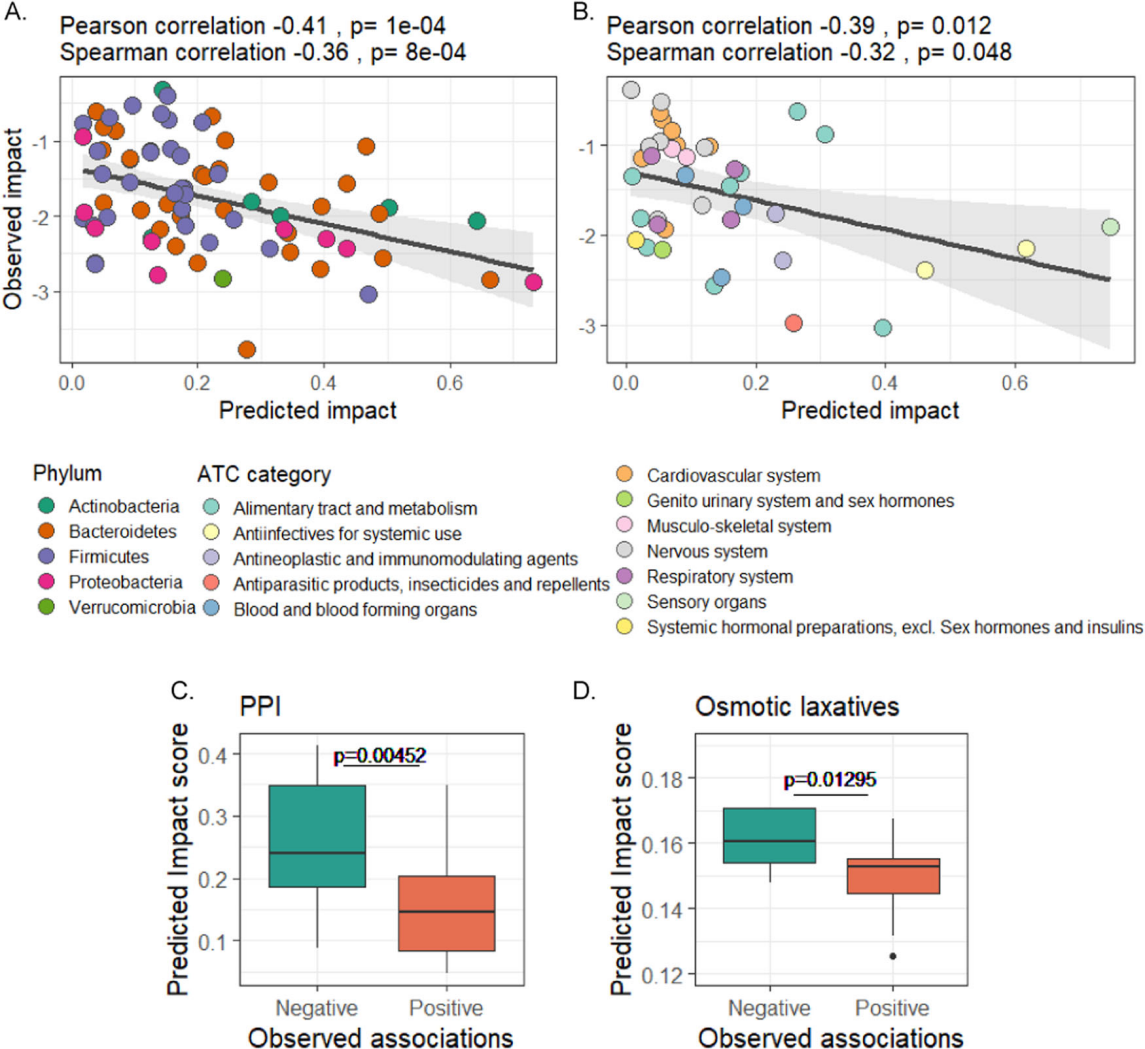
Comparison between model predictions and drug-induced dysbiosis in the Lifelines cohort. **A** Correlation between the average predicted impact per microbial genus and the taxa-averaged observed negative impact in the Lifelines cohort. (*n* = 84 taxa; error bands show standard error) **B** Correlation between the average predicted impact per drug group and drug-averaged observed impact in the Lifelines cohort. (*n* = 40 drugs; error bands show standard error) **C**, **D** Differences in predicted impact scores between taxa with positive vs negative association with two common drug groups, proton-pump inhibitors (**C**, Two-sided Wilcoxon rank-sum test, effect size 0.425, *n* = 66 taxa) and osmotic laxatives (**D**, Two-sided Wilcoxon rank-sum test, effect size = 0.466, *n* = 45 taxa). A line across the box indicates the median. The whiskers are lines extending from Q1 and Q3 to endpoints that are defined as the most extreme data points within Q1 − 1.5 × IQR and Q3 + 1.5 × IQR, respectively.

To this end, we obtained summary statistics data from the Lifelines Dutch microbiome project, which comprehensively characterized the gut microbiome of 8202 individuals along several clinical phenotypes^38^ Specifically, among the data publicly available for this project, is a list of identified negative and positive associations between drugs and microbes, allowing us to compare our model’s predictions concerning the negative impact of drugs on microbial growth to observed patterns across this cohort (measured by the treatment effect size, see Methods). We found a moderate, yet statistically significant agreement between our model and these data (Fig. 5), with taxa predicted by our model to be more drug-sensitive exhibiting strong negative effect by drugs across Lifelines individuals (Pearson correlation −0.41, *p* = 1 × 10^−4^, Spearman correlation −0.39, *p* = 8 × 10^−4^; Fig. 5A), and drugs predicted to have a strong impact on average by our model, showing a strong impact on microbes’ abundances (Pearson correlation −0.32, *p* = 0.012, Spearman correlation −0.39, *p* = 0.048; Fig. 5B). This suggests that our model correctly identifies taxa that are more drug-sensitive and drugs that have a more pronounced modulation of the microbiome.

Lastly, we focused on drugs that cause particularly drastic microbiome perturbation (associated with the abundance of >30 taxa in the Lifelines dataset) and examined whether our model can distinguish taxa resistant to these drugs (and hence exhibit positive association with the drug in the Lifelines cohort) vs. those that are sensitive to the drug and exhibit negative association. In two drug groups, proton-pump inhibitors and osmotic laxatives, we identified this expected statistically significant difference, with markedly higher predicted impact scores in taxa with a negative association with these drugs compared to taxa with positive association (Wilcoxon rank-sum test; Fig. 5C, D). Taken together, the above results from both a population-wide cohort and longitudinal studies demonstrate the model’s in vivo applicability.

### Characterizing links between adverse drug reactions and anti-commensal activity

**Fig. 6:**
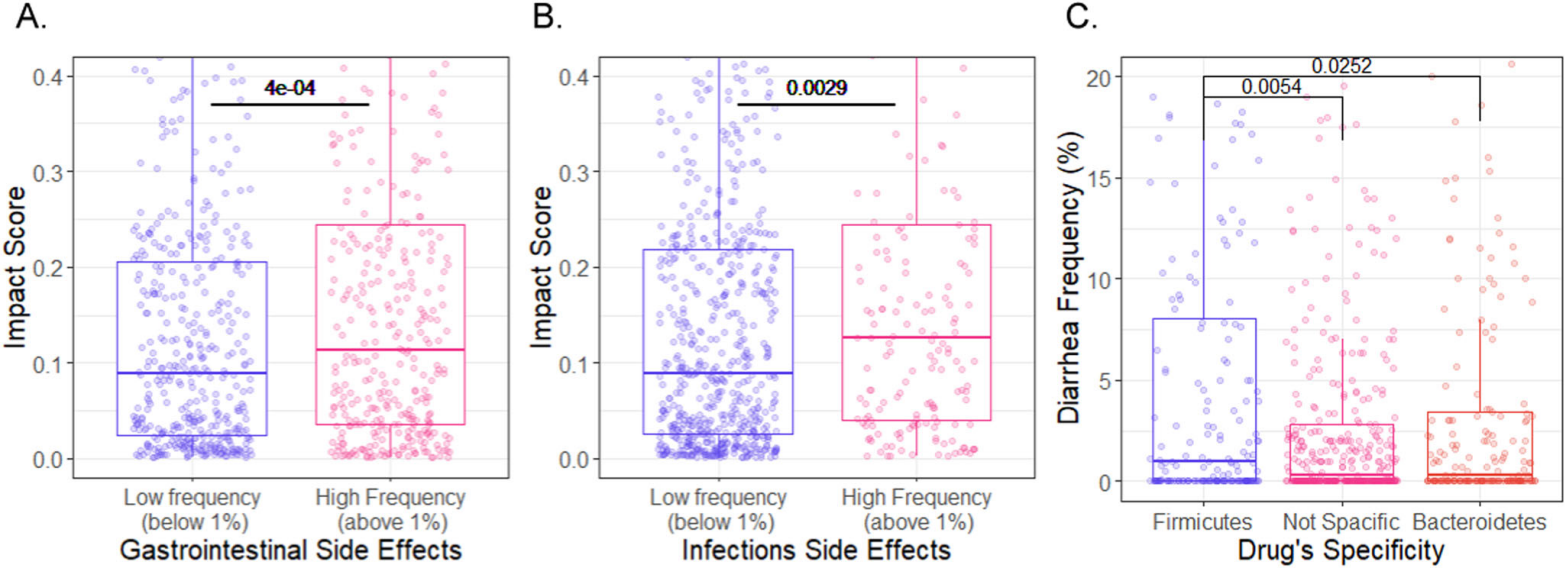
Association of anti-commensal activity and adverse drug reactions. Box plots illustrate the difference in predicted impact scores between drugs with **A** high vs. low frequency of gastrointestinal drug adverse effects and **B** high vs. low frequency of infectious drug adverse effects. **C** Difference in diarrhea frequency between drugs with impact specificity to *Firmicutes* and *Bacteroidetes*, as well as drugs without specificity to any phyla (*n* = 771 drugs, two-sided Wilcoxon rank-sum test). A line across the box indicates the median. The whiskers are lines extending from Q1 and Q3 to endpoints that are defined as the most extreme data points within Q1 − 1.5 × IQR and Q3 + 1.5 × IQR, respectively.

Lastly, we examined whether the antimicrobial properties of drugs may be related to their side effects. Specifically, following previous studies that highlighted a link between drug-induced dysbiosis and side effects in a handful of medications, we wondered whether using our model’s predictions we can observe this link on a much larger scale. To this aim, we collected and curated side effects reports from the Side Effect Resource (SIDER) database^39^ for all non-antibiotic small-molecule drugs (*n* = 771 compounds). Then, we predicted the impact of each of these drugs on the representative microbiome community described above and calculated the mean impact score of each drug across all taxa. Comparing the drugs’ impact scores with their cataloged side effects, we found that both gastrointestinal and infection-related adverse effects were strongly associated with the drug’s impact on the microbiome. Specifically, non-antibiotic medications with a high incidence of these side effects exhibited a significantly higher impact score compared to those with a low incidence of these side effects (Fig. 6A, B), suggesting that perhaps similarly to antibiotics^11^, drugs with an extensive impact on the intestinal community might facilitate gastrointestinal side effects and colonization of pathogenic bacteria.

Based on this finding, we next examined whether specific dysbiosis patterns might be associated with various adverse effects. Above, we found that each drug tends to affect mostly strains for one of the two main phyla of the human microbiome, *Firmicutes* or *Bacteroidetes*, with a smaller effect on strains from the other. Here, we compared for each drug the difference in its impact on these two phyla (as a measure of its specificity) and calculated the association between each side effect and this drug’s specificity. We found that diarrhea is significantly more common in patients receiving drugs that are more harmful to *Firmicutes* than to *Bacteroidetes*, with a ~66% increase in incidence rate (ANOVA, *p* = 5 × 10^−3^, Fig. 6C). Interestingly, in line with our findings, stool consistency was positively associated with *Bacteroidetes*: Firmicutes ratio in a healthy woman population^40^. Based on those findings, we hypothesize that the mode of drug-induced dysbiosis may explain the patterns of certain adverse effects.

## Discussion

In this study, we developed a machine-learning approach that integrates chemical properties and genomic content to predict the impact of drugs on microbiome taxa. We demonstrated the utility of this approach in a range of in vitro and in vivo settings, from pairwise microbe–drug experiments, through animal models, to clinical trials. We specifically focused on mouse and human longitudinal data to support our findings. This analysis design enables direct evaluation of the drug’s impact on intestinal bacteria, while minimizing other confounding factors that can impact case-control or population-wide studies. Importantly, beyond its ability to predict the impact of specific drugs on specific microbes, this approach uncovers the determinants behind the interactions between pharmaceuticals and microbes and systematically maps the interactions between every drug and all representative members of the human microbiome. Given this extensive large-scale catalog, we were able to further reveal strong associations between the anti-commensal properties of drugs and gastrointestinal side effects.

Throughout this work, we made a substantial effort to validate obtained predictions via experimental data using an extensive collection of in vitro experiments, longitudinal clinical studies, and large-scale population cohorts. Unfortunately, however, the current state of research limits our ability to perform a more comprehensive examination of computational predictions, as the number of microbe–drug interaction studies is still relatively low, and the number of studies that make such data (and specifically shotgun metagenomic data) publicly available for analysis is even more restricted. Furthermore, even when data were available, technical variations between studies often make comparison and validation challenging. For example, our attempt to benchmark our model’s predictive performances using data from a different lab experiment was complicated given the different approaches used (optical density vs mass spectrometry). Still, the fact that our model retained a reasonable predictive power in the face of such variation in methodologies indicates that it captures real, non-study- or method-specific aspects of drug–microbiome interactions. While the validation of many such interactions is an avenue for future studies, we believe that the translation of in vitro experiments to clinical settings using machine learning frameworks offers a powerful innovation.

Despite these advances, our approach is not without drawbacks and relies on several main simplifications. First, the anti-commensal effect of a given drug clearly depends on its bioavailability in the site of action, yet our model relies on data from a high-throughput screen in a fixed concentration. Although the authors demonstrated that this concentration is within an order of magnitude from the intestinal concentration for most drugs, it might be lower or higher for some compounds and could be influenced by both host and microbial factors. Furthermore, as our method and analysis here utilize a single strain as a representative of each species, it is important to note that it may overlook certain strain-specific variations in response to drugs. Such a variation may include factors with a well-documented influence on antimicrobial and anti-drug resistance, such as the presence of specific xenobiotic-metabolizing enzymes and multidrug resistance transporters^41,42^. While considering such strain-level resolution can be crucial for accurate prediction of drug response in various settings, the lack of available multi-strain data for model training currently limits our ability to incorporate these details. Nonetheless, as shown above, our model is capable of distinguishing higher-level phylogenetic patterns, while supporting potential future extensions to include strain-level resolution as more data become available. Lastly, rich ecological dynamics between microbiome community members and their interactions with epithelial and immune cells along the gastrointestinal tract could markedly alter the impact of various drugs on the microbiome^43^.

Importantly, while high-throughput experiments provide many important definitive insights into complex biological systems, machine learning methods can be used to complement such experiments and to allow researchers to further identify trends or extrapolate observed patterns. Even though future high-throughput screens will likely provide additional valuable information on drug–microbiome interactions, the scale of this space is already vast, and it is unlikely that experimental methods will be able to keep up with the rapid development of new compounds and with the pace at which new microbial strains are discovered and sequenced. Moreover, such in vitro experiments often require that assayed microbes be isolated and cultured, which is often challenging or not feasible, especially for gastrointestinal, host-associated species. We, therefore, believe that computational prediction of drug–microbe interactions is not only of value but a crucial component of future research on the interactions between drugs and the human microbiome, with numerous applications.

Looking forward, dissecting the plethora of interactions between drugs, microbes, and the host holds great promise for clinical applications^5,13^. For example, the ability to successfully predict drug–microbiome interactions in vivo can facilitate future efforts to predict microbiome-wide drug sensitivity across global, healthy populations, and for generating novel population-level hypotheses concerning drug–microbiome interactions. In the last decade, researchers have characterized the cardinal role of the human microbiome on pharmaceutical treatment, highlighting various processes such as biotransformation to inactive or even toxic compounds^44–46^, alternation of pharmacokinetics and pharmacodynamic properties^3^, and adverse reactions associated with drug-induced dysbiosis^47,48^. Excitingly, such observations are currently being translated into new clinical intervention protocols, optimizing treatment outcomes by developing inhibitors for drug-metabolizing enzymes^49–51^, and manipulating the microbial community using prebiotics and fecal microbiome transplants^52^. Our methodology could substantially complement these efforts, guiding future drug development attempts by providing crucial information about potential personal alterations to the microbiome following pharmaceutical treatment and identifying possible mechanistic explanations for the anti-commensal activity and side effects. The use of this and other computational tools^15^ could accordingly benefit future efforts in pharmacological and microbiome research, paving the way for personalized pharmaceutical therapy and tailored microbial interventions.

## Methods

### Machine-learning model, data, and evaluation

We implemented a machine learning model (see detailed below) to estimate the impact of each drug on each microbiome member. The model represents each drug–microbe pair as a vector of features. Drugs are described by a set of physical-chemical and structural properties, obtained from SMILES representation, using RdKit, an open-source chemoinformatic program^53^. Each microbe is described by its KEGG pathways’ scores based on its genomic content, calculated using a previously published method^54^. Briefly, the score of each KEGG pathway represents the number of KEGG orthology groups (KOs) in that pathway (with KOs associated with several pathways being partitioned between these pathways). The list of all drug and microbe features is available in Supplementary Data 1.

To train and validate our model, we utilized data previously published by ref. ^11^. In brief, the data describes an in vitro screen of 1197 compounds against 40 microbial strains. For 36 of the 40 tested microbial strains, we obtained KEGG KO annotation from the IMG GOLD database^55^. The genomes of three strains (*Ruminococcus gnavus VPI C7-9*, *Bacteroides fragilis enterotoxigenic 20656-2-1*, and *Ruminococcus torques VPI B2-51*), whose KO annotations were not available in IMG, were downloaded from NCBI and annotated using BlastKOALA^56^. We discarded one strain (*Clostridium perfringens C36*) from further analysis as its genome wasn’t publicly available. Out of the 1197 screened drugs, we matched 1066 compounds to the DrugBank database^27^ by mapping their IDs to stitch5 IDs and curating this mapping manually (unmatched drugs were mostly non-pharmaceuticals or veterinary drugs). A binary interaction score (“0”—no interaction; “1”—growth inhibition) was given to each microbe–drug pair according to the definition in ref. ^11^.

Given these data, we trained several ML models including random forest (RF), supported vector machine (SVM) with either polynomial or radial basis function (RBF) kernels, and three regularized logistic regression models (ridge, lasso, and elastic net) with default hyperparameter. We intentionally focused on relatively simple and commonly used models to avoid the risk of overfitting. The performance of each model was estimated using tenfold cross-validation and evaluated by ROC AUC and PR AUC. The above models were implemented in R version 4.0.2^57^, using “tidymodels” package suite^58^ (version 0.14). Specifically, we used “ranger” for the RF model (version 0.13.1)^59^, “keranlab” (version 0.9-30) for SVM algorithms^60^, and “glmnet” (version 4.1-4) for logistic regression models^61^. The model’s performances were visualized using ggplot2 (version 3.3.5).

Evaluation of model performance on new microbes and new drugs was carried out using a leave-one-out cross-validation strategy. To determine how phylogenic and molecular similarities affect new microbe and drug predictions, we used phylogenic distance based on the 16S rRNA gene obtained via the Qiime2 fragment insertion plugin^62^ and molecular similarity based on Tanimoto coefficients on RDKit fingerprints.

To verify the robustness of various model predictions we conducted extensive analysis, comparing the machine learning techniques listed above to various naïve models and controlling for statistical noise (see full details in Supplementary Text 1).

### Feature importance

We calculated model feature importance using the method published by ref. ^17^, using the implementation in the “ranger” package^59^. This approach allows the calculation of importance scores with statistical significance measures. To estimate the per-strain feature importance we trained the RF model with a single strain at a time and extracted the feature importance using the above method. To illustrate strain-specific differences in feature importance score, we use a principal component analysis (PCA) and demonstrated statistically significant clustering by PERMANOVA (using the “vegan” package^63^, version 2.6-2).

### Metagenomic data pre-processing

We acquired 16S rRNA amplicon sequencing data from three published studies with metagenomic longitudinal sampling obtained before and after drug treatment^35–37^, as well as from a healthy western population cohort^28^. For consistency, we processed and analyzed each dataset in a similar way. Specifically, we obtained raw fastq files from public repositories (NCBI Sequence Read Archive or European Nucleotide Archive) and processed these data using Qiime2 version 2019-1^64^. We demultiplexed the data using the Qiime2 demux plugin, applied DADA2^65^ to denoise the data, and trimmed reads in each dataset to the first position with a median quality score under 30. Since the reverse reads were of low quality, these reads were discarded. To assign ASVs to taxonomy, we trained a Naive Bayes classifier per dataset using Qiime2’s feature-classifier plugin^66^. Classifiers were trained on reads extracted from the SILVA 99-OTU database^67^, according to the specific 16S hypervariable region used in each dataset. In each dataset, we removed samples with less than 1000 reads. We further removed rare and low abundance taxa, leaving those with abundance >0.5% in at least 0.5% of the samples. Read counts were normalized to sum to 1 within each sample, resulting in a table of relative abundances. When multiple timepoints were available, we averaged all before or after samples. Lastly, we calculated the change in relative abundance after treatment for each ASV using t-statistics.

### Predicting drug–microbe interactions

To predict a rich landscape of interactions between drugs and microbes, we processed metagenomic reads from a representative healthy western population (*n* = 90, mean age = 28)^28^. We used a single random metagenomic sample from each donor. We included in the analysis all ASV’s that appear in relative abundance above 0.5% resulting in 409 taxa in total. In parallel, we extracted from drugbank SMILES representations of all organic small-molecule drugs with ATC annotations (indicating that the drug is indeed in clinical use). To maintain the credibility of predictions, we focused on small-molecule drugs, removing proteins and inorganic compounds. Lastly, we trained our model on the full in vitro database and predicted the new interactions.

### Identifying protein of targets for human drugs

We extracted known, manually curated, protein targets of drugs from the drugbank database. We then mapped those proteins to KEGG KOs via the Uniprot ID and catalogs the taxa that encode these KO in their genome. Lastly, we compared the impact score of taxa with and without the presence of the KO using the Wilcoxon rank-sum test. Although this is not necessarily an accurate measure of structural identity, it reflects the overall functional similarity between the proteins.

### In vivo prediction

As before, the impact of each drug on each microbiome member was predicted using our model. The drug features were calculated as described above. The microbial features for each ASV were obtained from intermediate files of PICRUST2 that list KEGG KO annotation estimation^68^. Based on the full in vitro data, the model predicted the sensitivity of each ASV in the sample (i.e., the impact of the given drug on that ASV, normalized in a range of 0 to 1). We compared the model scores for ASV with decreased relative abundance (negative t-statistics) vs. those with increased relative abundance (positive t-statistics) by the Wilcoxon rank-sum test. To further verify that the results cannot be attributed to statistical noise, we repeated this analysis on randomly shuffled data.

### Analysis of lifelines data

Since raw data from this cohort is not publicly available, we utilized available summary statistics from the latest Lifelines publication^38^. Briefly, among other analyses, this publication applied a linear regression model (adjusting for confounding factors such as sex, BMI, and technical variables) to determine the impact of various drugs on microbiome composition. Following this analysis, they provided a list of identified negative and positive significant associations (with FDR-corrected *p* values <0.1) between microbes and drugs, along with a measure of drug impact (i.e., effect size). For our analysis, we calculated the observed impact of each drug in this cohort as the mean negative effect size across all microbes, and the observed microbial sensitivity of each taxon in this cohort, as the mean negative effect size across all drugs. As associations were frequently reported for a grouped drug class or higher taxonomical levels, we averaged the predicted impact score across all members of the group (for example, across several drugs in the given ATC code). For drugs with >30 drug-taxon associations, we compared the predicted impact score between microbes with negative and position effect sizes using the Wilcoxon rank-sum test.

### Associations between side effects and drug impact

We retrieved drug side effects from the SIDER database^39^. We selected side effects according to the MedDRA preferred term and all drugs with ATC annotation. Rare side effects that have been reported for less than 50 drugs were discarded. For each drug, we calculated side effect incidence frequency based on the mean frequency across all the data sources in the database and subtracted the incidence frequency of the placebo-treated groups. We grouped side effects of interest into several broader categories. Specifically, we grouped constipation, abdominal pain, diarrhea, gastrointestinal disorder, gastrointestinal pain, vomiting, nausea, abdominal discomfort, dyspepsia, flatulence, and abdominal pain upper into “Gastrointestinal side effects”. Similarly, we grouped infection, urinary tract infection, pneumonia, stomatitis, and upper respiratory tract infection into “Infection side effects”. We next, calculated the mean frequency across all side effects within each category. We then calculated the mean impact score of each drug across all microbes and compared this mean impact score for drugs above vs. below 1% side effect frequency.

Lastly, we explored the association between the dysbiosis pattern of drugs and their side effects. We calculated for each drug the difference in impact score between Bacteroidetes and Firmicutes. Based on this measure, we partitioned the drugs into four quartiles. Drugs in the fourth quartile were classified as firmicutes specific, drugs in the first quartile were classified as bacteroidetes specific, and drugs in the middle two quartiles were classified as not specific to any phyla.

### Statistics and reproducibility

Statistical calculations were conducted in R (version 4.0.2). We have used the “tidyveres” package for general data handling and cleaning (version 2.00), visualized results using “ggplot2” (version 3.3.5), and conducted statistical calculations using “vegan” (version 2.6-2). Since this work relies on previously published and available data, no statistical method was used to predetermine the sample size, no data were excluded from the analyses, and the experiments were not randomized and the investigators were not blinded to allocation during experiments and outcome assessment.

### Reporting summary

Further information on research design is available in the Nature Portfolio Reporting Summary linked to this article.

## Supplementary information

Supplementary Information

Description of Additional Supplementary Files

Supplementary Dataset 1-6

Reporting Summary

## Data Availability

In this work, we relied on previously published and publicly available datasets. Specifically, we collected SMILES representations, ATC classification, and recognized protein targets from the Drugbank database (version April 2020, ref. ^27^, https://go.drugbank.com/). Similarly, to describe specific microbial strains, we downloaded the KEGG KO annotation of 36 genomes from IMG GOLD (ref. ^55^, https://img.jgi.doe.gov/). Further, three strains whose KO annotations were not available in IMG were downloaded manually from NCBI Sequence Read Archive as further described in the Methods section, using accession codes ASM169987v1 (https://www.ncbi.nlm.nih.gov/assembly/GCF_001699875.1/), ASM983137v1 (https://www.ncbi.nlm.nih.gov/assembly/GCF_009831375.1/) and ASM15392v1 (https://www.ncbi.nlm.nih.gov/assembly/GCF_000153925.1/). To train and validate our in vitro machine learning model, we used supplementary information data previously published by ref. ^11^ and by ref. ^12^. To describe the community structure in a healthy human population, we downloaded raw sequences from ref. ^28^. We further downloaded from NCBI SRA and/or European Nucleotide Archive raw sequencing data to predict drug impact in vivo as described in Supplementary Data 4 refs. ^35–37^. We accessed summary statistics data from the Lifelines Dutch microbiome project as published in the Supplementary Data in ref. ^38^. Lastly, we obtained adverse effect information from the SIDER database (ref. ^39^, SIDER version 4.1, http://sideeffects.embl.de/).

## Code availability

We used RDKit with Python API to calculate chemoinformatic properties and molecular similarity (https://www.rdkit.org/) and BlastKOALA (https://www.kegg.jp/blastkoala/) to annotate genomes according to KEGG KOs.

Raw metagenomic data were processed by qiime2 (https://qiime2.org/, version 2019-1), and denoised by DADA2. Similarly, the qiime2 fragment insertion algorithm was used to calculate phylogenetic relationships and PICRUST2 was used to predict functional abundance per ASV.

Data analysis was conducted in R version 4.02 using the packages “tidyveres” (version 2.00, general data handling and cleaning), “tidymodels” (version 0.14, machine learning pipelines), “ranger” (version 0.13.1, RF model), “keranlab” (version 0.9-30, SVM algorithms), “glmnet” (version 4.1-4, logistic regression models), vegan (version 2.6-2, statistical analysis), and ggplot2 (version 3.3.5, visualization).

Custom analysis code used in this study can be found on GitHub at: https://github.com/borenstein-lab/drug_microbiome.
