# Supplementary material for: A data-driven approach for predicting the impact of drugs on the human microbiome
Source: Nat Commun. 2023 Jun 17;14:3614. doi: 10.1038/s41467-023-39264-0 (PMC10276880; doi:10.1038/s41467-023-39264-0)
Supplement: Supplementary file 1 — Supplementary Information [file 41467_2023_39264_MOESM1_ESM.pdf]

# Supplementary Information

Supplementary information for “A data-driven approach for predicting the impact of drugs on the human microbiome”

## Supplementary Figures

Supplementary Figure S1 – Variance in model performance across 100 10-fold cross-validation iterations.

Supplementary Figure S2 –Validation against additional in-vitro datasets

Supplementary Figure S3 – Differences in the importance of drug features between different microbial taxa.

Supplementary Figure S4 – Differences in drug sensitivity in genus scale Supplementary Supplementary Figure S5 – Controls for in-vivo model predictions.

## Supplementary Table

Supplementary Table 1 – A summary of in-vivo studies used in our analysis.

## Supplementary Text

Supplementary Text 1 – Machine learning model additional evaluation.

Supplementary Text 2 – Suggested microbial targets of human-targeted drugs.

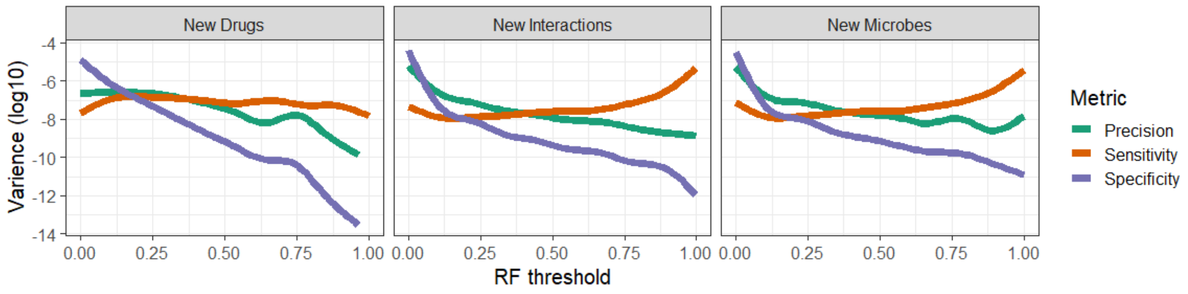


**Supplementary Figure S1 – Variance in model performance across 100 10-fold cross-validation iterations.** The calculated variance in the precision, sensitivity, and specificity, as a function of the RF thresholds used is presented for the three learning tasks: new drugs, new interactions, and new microbes.

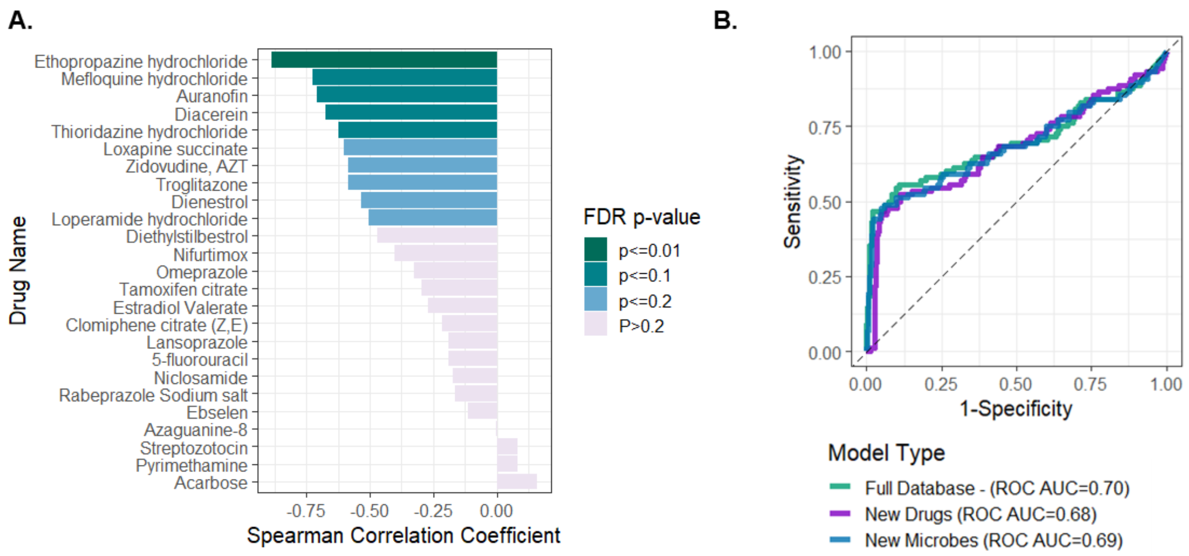


**Supplementary Figure S2 –Validation against additional in-vitro datasets. A**. Correlation between the model's predicted impact scores and in-vitro determined IC25 measurements. Experiments were conducted on a range of microbial strains (16 drugs against 12 microbes, 6 drugs against 16 microbes, and 3 drugs against 25 microbes). Exact FDR-corrected p-values are available in Supplementary Data 2 **B**. Receiver operating characteristic (ROC) curve for independent predictions of 43 drugs against 19 microbes in three prediction settings: full dataset, new microbes, and new drugs.

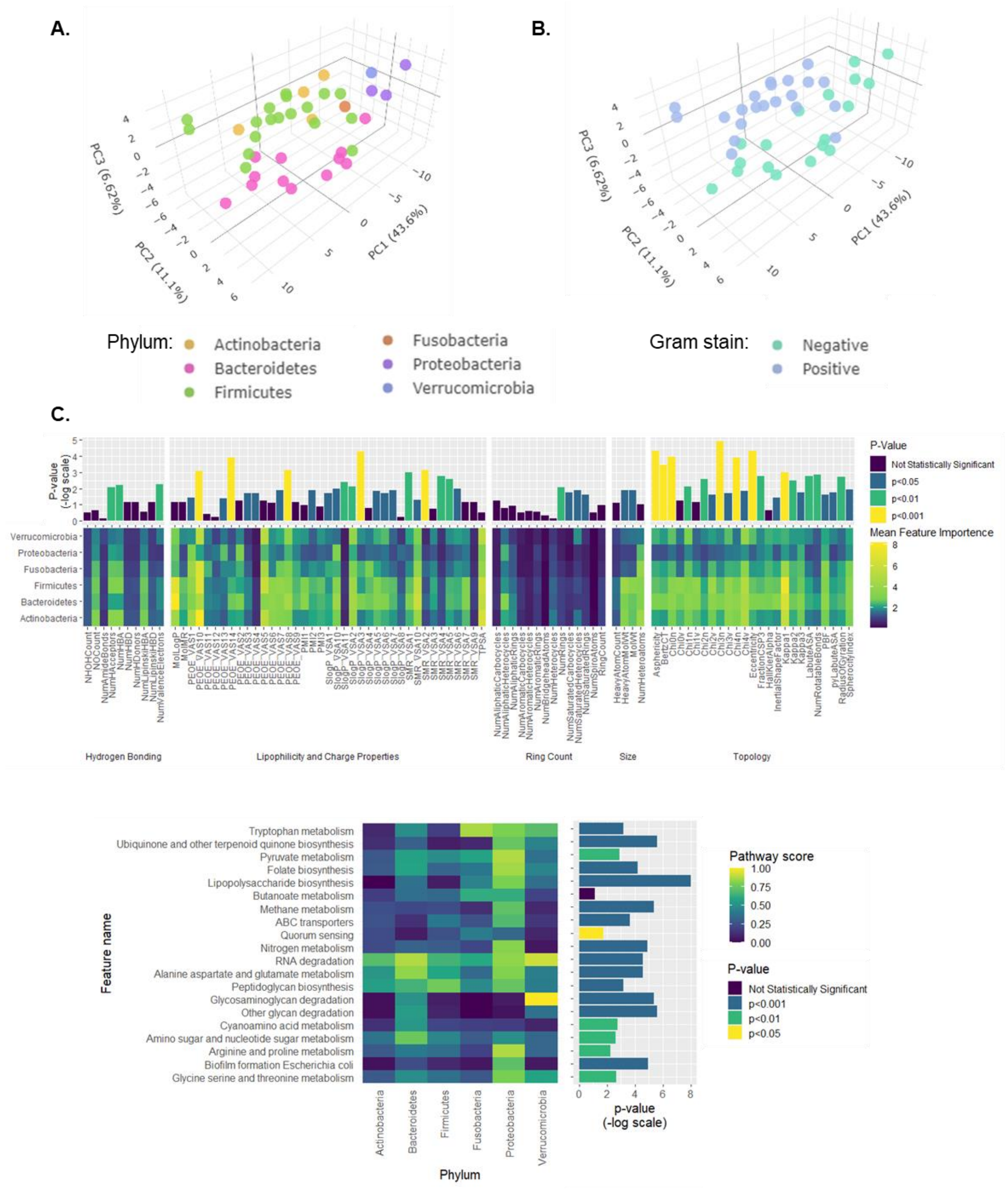


**Supplementary Figure S3 – Differences in the importance of drug features between different microbial taxa**. Principal Component Analysis (PCA) of the importance of drug features. Each point represents a single microbial strain and is clustered according to **A** – Phyla (PERMANOVA, p = 0.001) and, **B** – Gram stain (PERMANOVA, p = 0.017). **C** – Differences in mean feature importance between phyla. Top panel – Two-way ANOVA FDR corrected p-value (in -$\log_{10}$ scale) for each feature. Bottom panel – Mean feature importance for each phylum. **D** - Comparison between the abundance of microbial features across phyla. The heat-map on the left shows the mean abundance score of the top 20 microbial features across phyla. The bar plot on the right shows the FDR corrected p-values of the Kruskal-Wallis test (in -log10 scale).

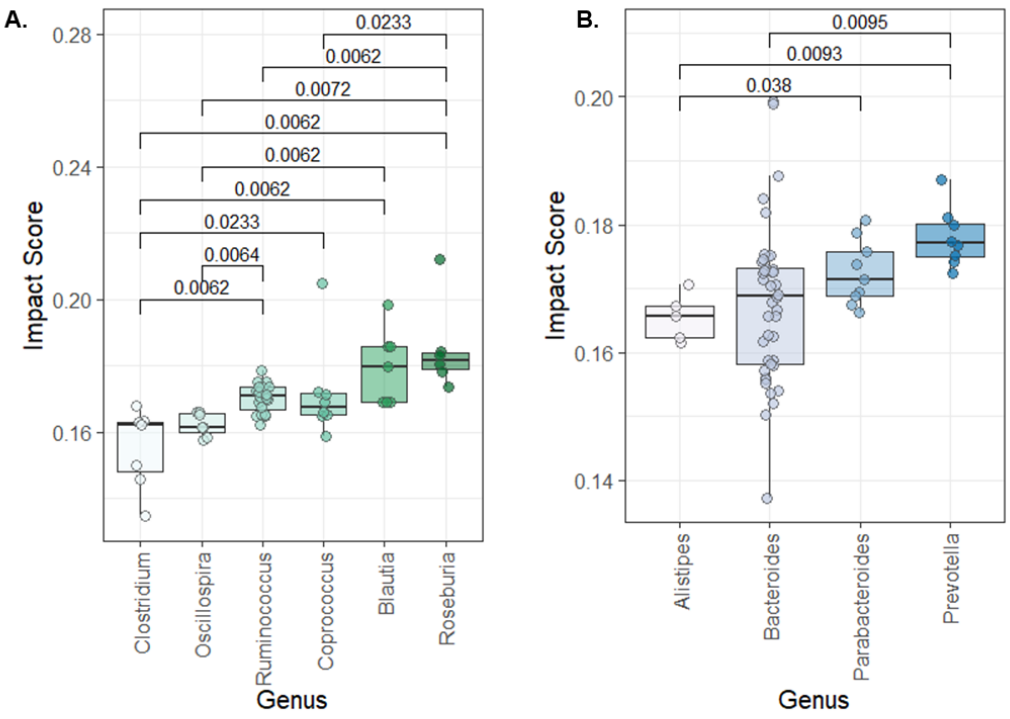


**Supplementary Figure S4 – Differences in drug sensitivity in genus scale**. Differences in drug sensitivity between genus-level members of the phyla **(A)** Firmicutes (n= 55 taxa) and **(B)** Bacteroidetes (n = 64 taxa, Wilcoxon rank sum test; FDR corrected p values). A line across the box indicates the median. The whiskers are lines extending from Q1 and Q3 to endpoints that are defined as the most extreme data points within Q1 − 1.5 × IQR and Q3 + 1.5 × IQR, respectively.

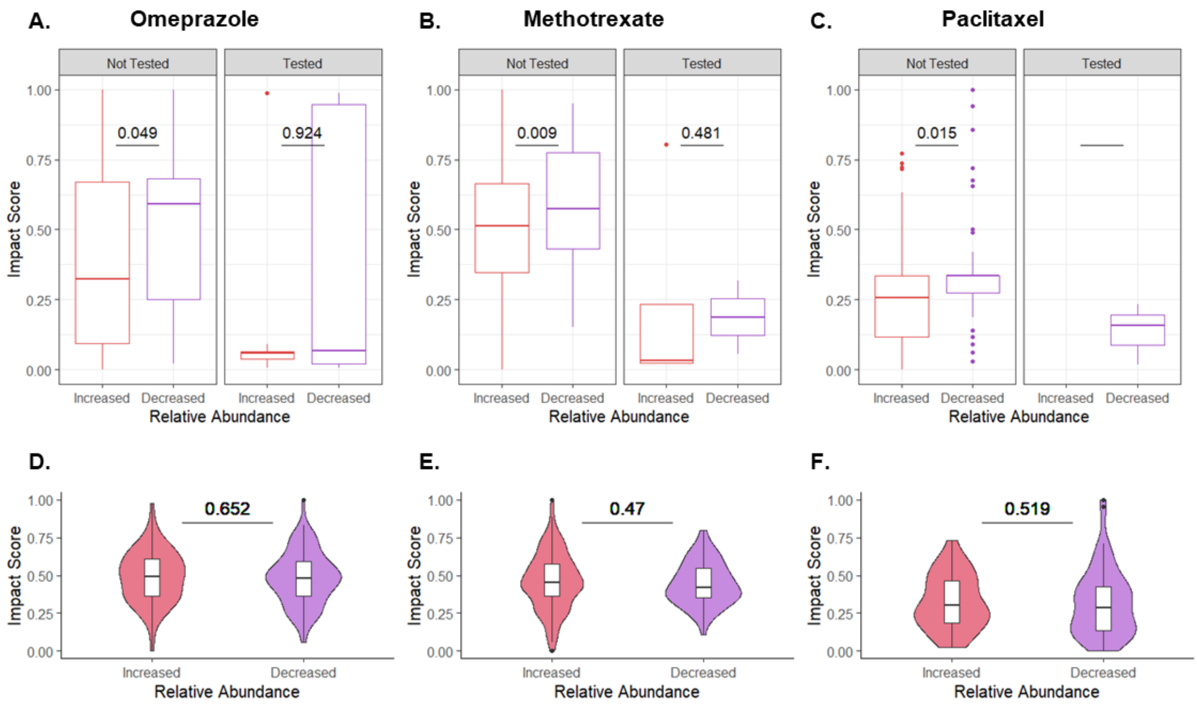


**Supplementary Figure S5 – Controls for in-vivo model predictions. A-C –** Comparison in prediction scores between taxa with increased and decreased abundance after drug administration. Results are shown separately for taxa present and absent from the in-vitro dataset. **D-E –** Comparison in prediction scores between taxa with increased and decrease abundance following drug administration using a model with shuffled features. Statistical significance was computed using the Wilcoxon rank-sum test. (Sample size, A,D – 153 taxa, B,E – 120 taxa, C,F – 149 taxa). A line across the box indicates the median. The whiskers are lines extending from Q1 and Q3 to endpoints that are defined as the most extreme data points within Q1 − 1.5 × IQR and Q3 + 1.5 × IQR, respectively.

| Study name | Host | Drug treatment | Sample size | Sequencing method | DOI |
|---|---|---|---|---|---|
| Prolonged use of a proton pump inhibitor reduces microbial diversity: implications for Clostridium difficile susceptibility | Humans | Omeprazole | 10 | 16s V3-V5 | 10.1186/2049-2618-2-42 |
| A Two-Way Interaction between Methotrexate and the Gut Microbiota of Male Sprague–Dawley Rats | Male Sprague–Dawley rats | Methotrexate | 22 | 16s V1-V2 | 10.1021/acs.jproteome.0c00230 |
| Dominant Role of the Gut Microbiota in Chemotherapy Induced Neuropathic Pain | Mice | Paclitaxel | 32 | 16s V4-V5 | 10.1038/s41598-019-56832-x |

**Supplementary Table 1 –** A summary of in-vivo studies used in our analysis.

# Supplementary Text 1 - Machine learning model additional evaluation

We have performed additional analyses to confirm the robustness of our approach, as described below. First, we compared the performance of the random forest model used in our framework to several other machine learning methods, including supporting vector machines with polynomial or radial basis function kernels, ridge logistic regression, elastic net logistic regression, and lasso logistic regression. As evident from the figure below (Panel A), our random forest model outperformed all other methods in predicting new drug-microbe interactions. All models were run with default parameters and evaluated using the same 10-fold cross-validation scheme. Notably, further hyper-parameter tuning did not improve the overall performance of our random forest model.

We further validated that the successful predictions obtained by our model cannot be explained by statistical noise or other artifacts in the data by randomly shuffling the values within each feature. Across all prediction scenarios (prediction of new drug-microbe interactions, prediction of the impact on new microbes, and predicting the impact of new drugs), prediction performances were close to random when using the shuffled set of features (Panel B).

Lastly, we compared the performance of our random forest model with a naïve null model, estimating the probability for a new interaction based on the average interaction of that same drug (or microbe) with other microbes (or drugs) and using the same sampling procedure as in the random forest model. Put differently, to predict the impact of a new drug, the data is partitions using a leave-one-drug-out scheme, and then the impact score of that drug on a given microbe is calculated based on the fraction of other drugs that impact this microbe (Panel C). Similarly, to predict the impact of drugs on a new microbe, the data is portioned by the leave-one-microbe-out approach, and then the impact score of a given drug on that microbe is calculated by considering the fraction of all other microbes that this specific drug affects (Panel D). Lastly, to predict new drug-microbe interactions the data is partitioned into ten bins, following the 10-fold cross-validation methodology, and then, within every bin, the impact score for all interactions is calculated based on the square-root multiplication between the fraction of drugs that impact a specific microbe and the fraction of

microbes that the drug impacts in all other 9 bins (Panel E). This analysis demonstrated that for predictions on new drugs (which is arguably the most interesting setting), the random forest model markedly outcompetes the naïve model. For new interactions and new microbes, the random forest model still outcompetes the naïve model (McNamar's test, $p<2.2*10^{-16}$), but notably, the improvement in performances is relatively very small. This is perhaps not surprising since most drugs tend to have a similar impact on most microbes. Yet, importantly, when focusing on the subset of drugs and microbes with a high level of variability (the top 20% observations with highest variance), the differences in performance between our random forest model and the naive model become noticeably more substantial.

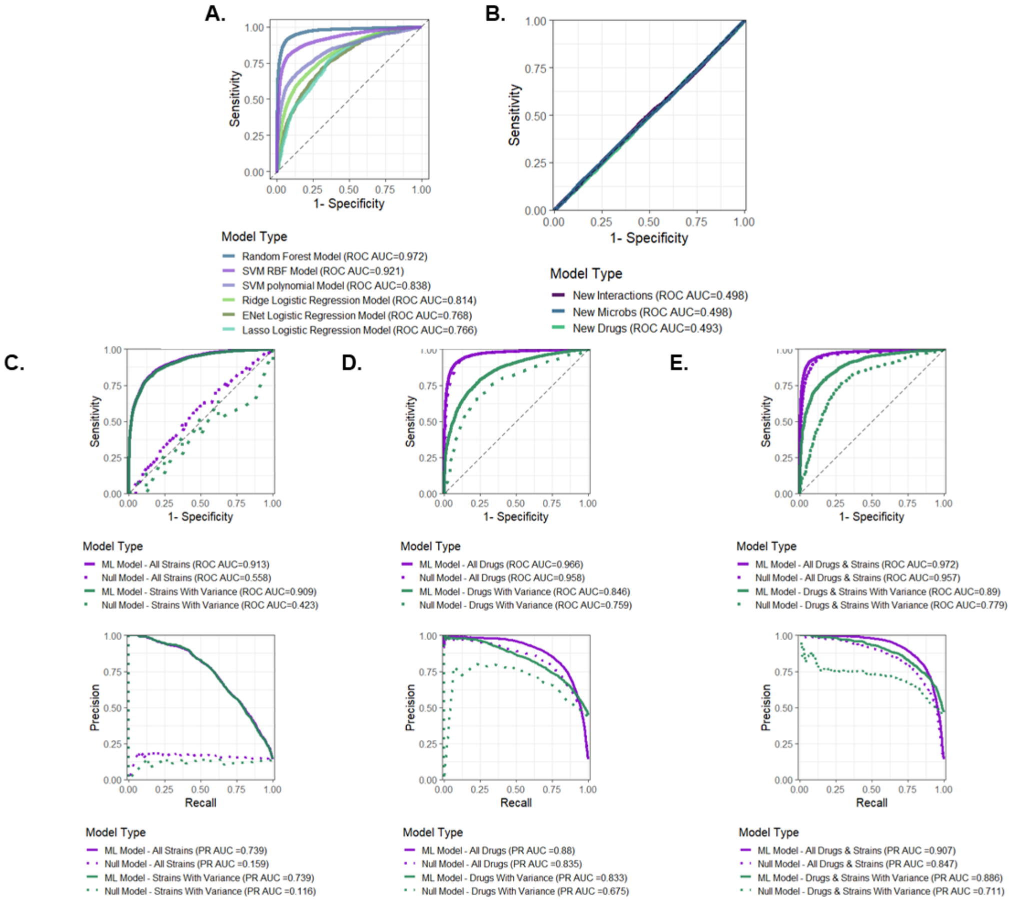


**Evaluation of the random forest models in comparison to other machine learning models and naïve predictions. A** – A compression between serval machine learning models in predicting new drug-microbe interactions. Abbreviations - Elastic Net (ENet), supporting vector machine radial basis function kernel (SVM RBF), supporting vector machine polynomial kernel (SVM polynomial). **B** – Performances of the random forest model on a dataset with shuffled features across three learning scenario. **C-E** – An ROC curve and a PR curve of a compression between our machine learning model and a naïve null model in predicting interactions with a new drug (C), a new strain (D), and a new drug-microbe pair (E). Each plot represents predictions on the full dataset, as well as on the subset of the dataset with the highest variability (see Supplementary Text 1).

## Supplementary Text 2 - Suggested microbial targets of human-targeted drugs

In this section, we aimed to highlight potential future research that could be addressed using a computational approach as it has the potential to identify a possible mechanistic explanation for the anti-commensal properties of human-targeted drugs. Unlike antibiotics, most human-targeted medications do not have a recognized microbial target. For this purpose, we obtained manually curated protein target information from the DrugBank database and mapped these proteins to their microbial orthologs (see Methods). We found 899 drug-microbial target interactions between 497 compounds and 198 proteins. We then searched for drugs that had significantly different impact scores between taxa with and without the target protein, identifying 201 such pairs (Two-sided Wilcoxon rank-sum test, all comparisons with FDR $p < 0.05$, exact p-values in Supplementary Data 6). Among these are compounds with previously identified targets, such as nucleotide analogs which inhibit thymidylate biosynthesis, a proposed target for antibiotics[35], and streptozotocin, which inhibits intracellular protein glycosylation[36]. We further identified possible targets of compounds with characterized antimicrobial activity but without recognized microbial protein targets including, sevoflurane inhibition of calcium-transporting ATPase[36] and sitagliptin inhibition of Dipeptidyl peptidase 4 orthologues[37]. This analysis could pave the way for deeper understanding and possible prevention of this unwanted off-target activity.

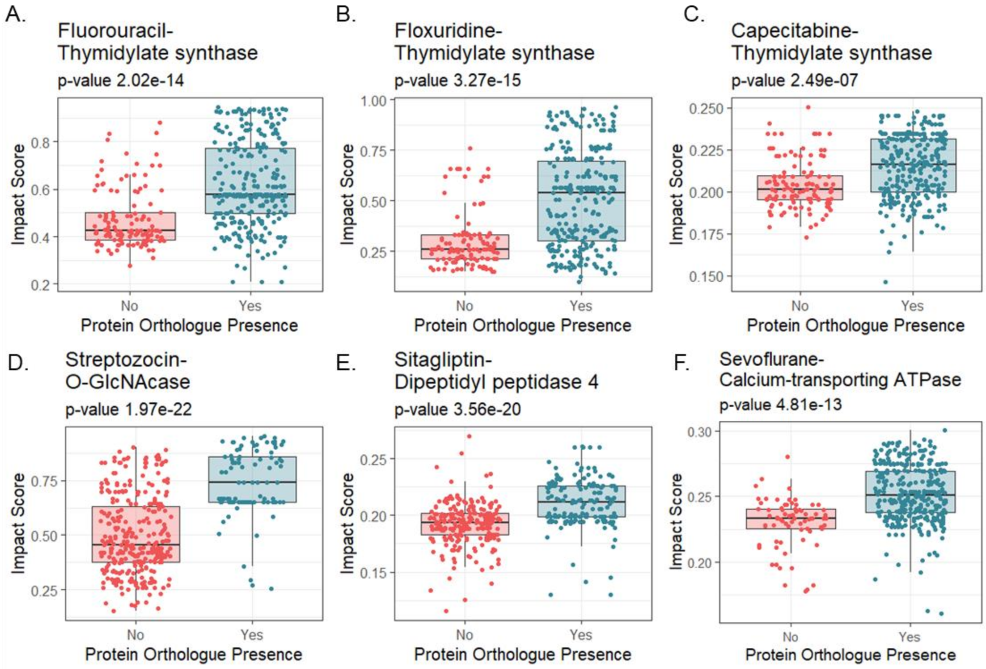


**Differences in impact score between taxa according to the presence of protein target orthologues**. **A-F** – Difference in impact score for Fluorouracil (A), Floxuridine (B), Capecitabine (C), Streotozocin (D), Sitagliptin (E), and Sevoflurane (F) between taxa with and without orthologue to thymidylate synthase (A), thymidylate synthase (B), thymidylate synthase (C), O-GlcNAcase (D), Dipeptidyl peptidase 4 (E), and calcium-transporting ATPase (F). n = 409 taxa,Significance was calculated using the two – sided Wilcoxon rank-sum test and FDR-corrected p-values. A line across the box indicates the median. The whiskers are lines extending from Q1 and Q3 to endpoints that are defined as the most extreme data points within Q1 − 1.5 × IQR and Q3 + 1.5 × IQR, respectively.
