# Supplementary material for: A data-driven approach for predicting the impact of drugs on the human microbiome
Source: Nat Commun. 2023 Jun 17;14:3614. doi: 10.1038/s41467-023-39264-0 (PMC10276880; doi:10.1038/s41467-023-39264-0)
Supplement: Supplementary file 2 — Description of Additional Supplementary Files [file 41467_2023_39264_MOESM2_ESM.pdf]

File Name: Supplementary Data 1

Description: A list of machine learning model features used in our framework (92 drug features and 148 microbial features).

File Name: Supplementary Data 2

Description: Correlation between the model's predicted impact scores and in-vitro determined IC25 measurements

File Name: Supplementary Data 3

Description: The model's drug and microbial feature importance p-values.

File Name: Supplementary Data 4

Description: A list of machine learning model features used in our framework (92 drug features and 148 microbial features).

File Name: Supplementary Data 5

Description: A catalog of interactions between 2,585 drugs and 409 taxa

File Name: Supplementary Data 6

Description: Possible protein targets of human-targeted drugs with statistical significance of the difference in impact scores.
