# Supplementary material for: A data-driven approach for predicting the impact of drugs on the human microbiome
Source: Nat Commun. 2023 Jun 17;14:3614. doi: 10.1038/s41467-023-39264-0 (PMC10276880; doi:10.1038/s41467-023-39264-0)
Supplement: Supplementary file 4 — Reporting Summary [file 41467_2023_39264_MOESM4_ESM.pdf]

nature portfolio

Corresponding author(s): Elhanan Borenstein

Last updated by author(s): May 14, 2023

Reporting Summary

Nature Portfolio wishes to improve the reproducibility of the work that we publish. This form provides structure for consistency and transparency in reporting. For further information on Nature Portfolio policies, see our Editorial Policies and the Editorial Policy Checklist.

Statistics

For all statistical analyses, confirm that the following items are present in the figure legend, table legend, main text, or Methods section.

n/a	Confirmed	
☐	☒	The exact sample size (*n*) for each experimental group/condition, given as a discrete number and unit of measurement
☐	☒	A statement on whether measurements were taken from distinct samples or whether the same sample was measured repeatedly
☐	☒	The statistical test(s) used AND whether they are one- or two-sided<br>*Only common tests should be described solely by name; describe more complex techniques in the Methods section.*
☐	☒	A description of all covariates tested
☐	☒	A description of any assumptions or corrections, such as tests of normality and adjustment for multiple comparisons
☐	☒	A full description of the statistical parameters including central tendency (e.g. means) or other basic estimates (e.g. regression coefficient) AND variation (e.g. standard deviation) or associated estimates of uncertainty (e.g. confidence intervals)
☐	☒	For null hypothesis testing, the test statistic (e.g. *F*, *t*, *r*) with confidence intervals, effect sizes, degrees of freedom and *P* value noted<br>*Give P values as exact values whenever suitable.*
☒	☐	For Bayesian analysis, information on the choice of priors and Markov chain Monte Carlo settings
☒	☐	For hierarchical and complex designs, identification of the appropriate level for tests and full reporting of outcomes
☐	☒	Estimates of effect sizes (e.g. Cohen's *d*, Pearson's *r*), indicating how they were calculated

Our web collection on statistics for biologists contains articles on many of the points above.

Software and code

Policy information about availability of computer code

Data collection	No software was used for data collection
Data analysis	We used RDKit with Python API to calculate chemo-informatic properties and molecular similarity (https://www.rdkit.org/) and BlastKOALA (https://www.kegg.jp/blastkoala/) to annotate genomes according to KEGG KOs.<br>Raw metagenomic data were processed by qiime2 (https://qiime2.org/, version 2019-1), and denoised by DADA2. Similarly, the qiime2 fragment insertion algorithm was used to calculate phylogenetic relationships and PICRUST2 was used to predict functional abundance per ASV.<br>Data analysis was conducted in R version 4.02 using the packages “tidyveres” (version 2.00, general data handling and cleaning), “tidymodels” (version 0.14, machine learning pipelines), “ranger” (version 0.13.1, RF model), “keranlab” (version 0.9-30, SVM algorithms), “glmnet” (version 4.1-4, logistic regression models), vegan (version 2.6-2, statistical analysis), and ggplot2 (version 3.3.5, visualization).<br>Custom analysis code used in this study was deposited to GitHub at: https://github.com/borenstein-lab/drug_microbiome.

For manuscripts utilizing custom algorithms or software that are central to the research but not yet described in published literature, software must be made available to editors and reviewers. We strongly encourage code deposition in a community repository (e.g. GitHub). See the Nature Portfolio guidelines for submitting code & software for further information.

Data

Policy information about availability of data

All manuscripts must include a data availability statement. This statement should provide the following information, where applicable:

- Accession codes, unique identifiers, or web links for publicly available datasets
- A description of any restrictions on data availability
- For clinical datasets or third party data, please ensure that the statement adheres to our policy

In this work, we relayed on previously publishes and publicly available datasets. Specifically, we collected SMILES representations, ATC classification, and recognized protein targets from the Drugbank database (version April 2020, reference 27, https://go.drugbank.com/). Similarly, to describe specific microbial strains, we downloaded the KEGG KO annotation of 36 genomes from IMG GOLD (reference 55, https://img.jgi.doe.gov/). Further, three strains whose KO annotations were not available in IMG were downloaded manually from NCBI Sequence Read Archive as further described in the Methods section, using accession codes ASM169987v1 (https://www.ncbi.nlm.nih.gov/assembly/GCF_001699875.1/), ASM983137v1 (https://www.ncbi.nlm.nih.gov/assembly/GCF_009831375.1/) and ASM15392v1 (https://www.ncbi.nlm.nih.gov/assembly/GCF_000153925.1/). To train and validate our in-vitro machine learning model, we used supplementary information data previously published by Maier et al. (reference 11) and by Li, L. et al. (reference 12). To describe the community structure in a healthy human population we downloaded raw sequences from Poyet, M. et al. (reference 28). We further downloaded from NCBI SRA and/or European Nucleotide Archive raw sequencing data to predict drug impact in-vivo as described in Supplementary Table 4 (references 35-37). We accessed summary statistics data from the Lifelines Dutch microbiome project as published in the supplementary tables in reference 38. Lastly, we obtained adverse effect information from the SIDER database (reference 39, SIDER version 4.1, http://sideeffects.embl.de/).

Research involving human participants, their data, or biological material

Policy information about studies with human participants or human data. See also policy information about sex, gender (identity/presentation), and sexual orientation and race, ethnicity and racism.

Reporting on sex and gender	The temporal analysis of the Omeprazole clinical trial was based on publicly available data as published in reference 35. As the analysis was conducted on a per-subject bases, sex was not included as a covariate. Analysis of drug-modulated microbiome perturbations in the Lifelines cohort was conducted based on summary statistics and hence sex information was not available. Side effect information was retrieved from the SIDER database and doesn't include sex stratification.
Reporting on race, ethnicity, or other socially relevant groupings	Our analysis is limited to publicly available data where race, ethnicity, or other socially relevant groupings were not available.
Population characteristics	We analyzed publicly available data from previously published papers. No new human data has been collected or generated for this study. For a full description of the cohorts used in our research, please refer to the original publications. Specifically, for further information on Omeprazole clinical trial see reference 35, for Lifelines cohort see reference 38, for healthy human population see reference 28, and for SIDER side effect resource see reference 39.
Recruitment	We analyzed publicly available data from previously published papers. No new human data has been collected or generated for this study.
Ethics oversight	NA. We analyzed publicly available data from previously published papers. No new human data has been collected or generated for this study.

Note that full information on the approval of the study protocol must also be provided in the manuscript.

Field-specific reporting

Please select the one below that is the best fit for your research. If you are not sure, read the appropriate sections before making your selection.

☒ Life sciences ☐ Behavioural & social sciences ☐ Ecological, evolutionary & environmental sciences

For a reference copy of the document with all sections, see nature.com/documents/nr-reporting-summary-flat.pdf

Life sciences study design

All studies must disclose on these points even when the disclosure is negative.

Sample size	Available data was searched using PubMed and Google Scholar. For in-vitro verification, we utilized data from two independent publications, Maier et al. (reference 11), which performed screening of 40 microbial strains against ~1200 compounds, and Li et al. (reference 12), which conducted a smaller screen of 43 drugs against 5 ex-vivo human fecal samples. Temporal in-vivo analysis was conducted on one human clinical trial and two animal models as described in Supplementary Table 1. As this analysis completely relied on publicly available data, no power analysis or sample size selection was done by the researchers.
Data exclusions	In the in-vitro analysis that was based on the data from Maier et al. (reference 11), we excluded one strain, Clostridium perfringens C36, from downstream analyses, as its genome wasn't publicly available. We similarly discarded any non-drug compounds or compounds that did not have available information on DrugBank.

Replication	Our model was extensively evaluated in three settings - new drug-microbiome interactions, new drugs, and new microbes. The new interactions settings were subjected to randomized 10-fold cross-validation, while the new drugs and new microbes settings were tested using a leave-one-out approach. We repeated each analysis 100 times to assess the robustness of our methods and to examine the variance between repeats. The models demonstrate minimal variance in precision, sensitivity, and specificity across iterations as shown in Supplementary Figure S1.
Randomization	We applied 100 iterations on each in-vitro new interaction, new drugs, and new microbes settings to carefully confirm that the results cannot be attributed to nonrandom splits. In each iteration the data allocation was random.
Blinding	Since data were collected, processed, and published initially as part of a previously published work, blinding during data collection is not relevant.

Reporting for specific materials, systems and methods

We require information from authors about some types of materials, experimental systems and methods used in many studies. Here, indicate whether each material, system or method listed is relevant to your study. If you are not sure if a list item applies to your research, read the appropriate section before selecting a response.

Materials & experimental systems

n/a	Involved in the study
☒	☐ Antibodies
☒	☐ Eukaryotic cell lines
☒	☐ Palaeontology and archaeology
☒	☐ Animals and other organisms
☒	☐ Clinical data
☒	☐ Dual use research of concern
☒	☐ Plants

Methods

n/a	Involved in the study
☒	☐ ChIP-seq
☒	☐ Flow cytometry
☒	☐ MRI-based neuroimaging
